# Collaborative Brain-Computer Interface for Aiding Decision-Making

**DOI:** 10.1371/journal.pone.0102693

**Published:** 2014-07-29

**Authors:** Riccardo Poli, Davide Valeriani, Caterina Cinel

**Affiliations:** Brain-Computer Interfaces Lab, School of Computer Science and Electronic Engineering, University of Essex, Colchester, United Kingdom; McGill University, Canada

## Abstract

We look at the possibility of integrating the percepts from multiple non-communicating observers as a means of achieving better joint perception and better group decisions. Our approach involves the combination of a brain-computer interface with human behavioural responses. To test ideas in controlled conditions, we asked observers to perform a simple matching task involving the rapid sequential presentation of pairs of visual patterns and the subsequent decision as whether the two patterns in a pair were the same or different. We recorded the response times of observers as well as a neural feature which predicts incorrect decisions and, thus, indirectly indicates the confidence of the decisions made by the observers. We then built a composite neuro-behavioural feature which optimally combines the two measures. For group decisions, we uses a majority rule and three rules which weigh the decisions of each observer based on response times and our neural and neuro-behavioural features. Results indicate that the integration of behavioural responses and neural features can significantly improve accuracy when compared with the majority rule. An analysis of event-related potentials indicates that substantial differences are present in the proximity of the response for correct and incorrect trials, further corroborating the idea of using hybrids of brain-computer interfaces and traditional strategies for improving decision making.

## Introduction

The human visual system is far superior to any automated computer system in the processing and interpretation of visual scenes in ordinary conditions. Nonetheless, there are many limitations in its ability to accurately perceive and interpret the external environment, particularly in the presence of complex scenes – where the perceptual load is high – in the absence of sufficient time to complete the visual parsing or when attention is divided amongst multiple tasks. The exact nature of the perceptual and cognitive limitations has been studied for decades and demonstrated, for example, by phenomena such as attentional blink, repetition blindness, illusory conjunctions and the ventriloquist effect, all showing how stimuli can be missed, perceived with the wrong features or mislocated [1]–[4]. Because of these, and many other limitations, observers can typically attend and accurately perceive only a subset of the features of a complex scene, thus affecting their ability to assess situations, which, in turn, may result in sub-optimal decisions.

These limitations can partly be overcome if two or more individuals are involved in the assessment process, as, naturally, a group of individuals has access to more information and can therefore produce better decisions than a single individual.

### Decision Making in Groups

Years of research on decision making have shown how group decisions can be superior compared to individual decisions in many different contexts (see, for example, [5]–[8]), including settings where individuals are involved in visuals tasks [9]. However, there are circumstances in which group decision-making can be disadvantageous [10], [11]. Flaws can be caused by, for example, difficulties in coordination and interaction between group members, reduced member effort within a group, strong leadership, group judgement biases, and so on [6], [8], [9].

Therefore, though typically optimal group decisions are mediated by communication and feedback, whereby members of a group share information and get to know other members' opinions [12], more communication and feedback is not necessarily better. A recent study [13], for example, has found that when there are time constraints or if leadership prevails, the process of combining information from freely-communicating individuals can be an obstacle to optimal decision-making.

Even when there is a group advantage, that does not always necessarily increase monotonically with the number of group members: the optimal group-size seems to depend on the task at hand [14].

Group decisions can be negatively affected by communication biases and group dynamics both in terms of the quality of the decisions and in terms of timing. This can be particularly true in circumstances where optimal decisions rely on accurate and rapid information about the external environment, and decisions have to be taken rapidly. In these circumstances, multiple individuals can provide more accurate information. However, at the same time, communication between those individuals and time pressure can deteriorate the quality of a decision and slow down the decision process.

### Neural Correlates of Decision Making and Collaborative Brain-Computer Interfaces

A way to bypass some of the disadvantages of group decisions – while at the same time preserving the advantages – is by exploiting neural information about the perceptual and cognitive processes related to decision making.

Neuro-imaging and other techniques can reveal important information about the different cognitive stages that lead to a decision. Early visual–evoked potentials, such as the P1 and N1, are sensitive to the level of attention of an individual engaged on a specific task, where, for example, the N1 amplitude decreases as the attentional level decreases [15], [16], while its latency is sensitive to the difficulty of the task. The difficulty of a task also affects amplitude and latency of the P300 [17], an event-related potential (ERP) associated to target detection and recognition. While these ERPs are typically associated with early perceptual and cognitive processing of events, there are other, later components, that are instead associated with decision processes preceding, for example, an overt response made by the observing individual. One of these is the Contingent Negative Variation, a slow ERP component related to the preparation for a motor response and stimulus anticipation. The amplitude of this component has been shown to be smaller before incorrect responses compared with correct responses [18]. The Error Related Negativity – an ERP component occurring about 50–80 ms after an incorrect response – can also provide information about levels of confidence of decision making as it is affected by certainty or uncertainty about own performance [19]. Further findings have shown how ERPs can be related to both conscious and unconscious error detection [20] and timing of decisions [21] – all factors that are relevant to efficient decision making.

Moreover, neural correlates of an individual decision can be detected as early as about 800 ms before an explicit response is given, as shown for example by [21]. We should also note that it has been known for a long time that other (behavioural) measurements, such as the response times, are influenced by, and thus can reveal, the confidence in a decision [22].

Given these psychophysiology findings, it would seem reasonable to attempt to exploit this information to improve decision making (e.g., decisions based on neural activity can potentially be faster or better). However, EEG data are too noisy to use neural correlates on their own to reliably provide information on (or aid) single decisions (all the previously mentioned reports base their findings on averaging the signals resulting from a large number of repetitions of each event).

Nonetheless, it is plausible to think that collective decisions could be based, or partly based, on the integration of the brain activity of the members of a group. In fact, bypassing overt interaction and communication between group members might help overcome some of the drawbacks of group decision-making, previously discussed, while still preserving a key benefit of group interactions: that individuals who are not very confident in their decisions will influence a group's decision less and *vice versa*. Group decision-making supported by the integration of neural activity would be particularly suitable – but not limited – to circumstances where decisions are based on a rapid and accurate assessment of the environment and where fast reactions are needed.

The possibility of aggregating the brain activity of members of a group to reach optimal decisions has been recently explored by [23], who integrated (offline) the EEG (electroencephalogram) activity of up to 20 individuals engaged in a simple perceptual decision-making task (i.e., discriminating between rapidly presented pictures of cars and faces). It was found that combining neural activity across brains of at least eight observers resulted in decisions more accurate than decisions based only on behavioural responses of single observers. Also, group decisions could be predicted not only by the neural activity related to the decision processes, but also by the neural activity correlated to early perceptual processing. This shows that, in the specific experimental settings of that study, “multi–brain” decisions can be taken faster than decisions based on overt communication (decisions could be made as early as 200 ms after stimulus presentation). However, accuracy of groups based on the integration of the members' neural signals was never superior to groups' behavioural performance. Also, in the study, classification was facilitated by the neural signals that are known to differentiate encoding of faces from encoding of other objects (different brain areas are known to process faces compared to object processing and face perception is known to produce very distinctive EEG signals, i.e., particularly the N170 ERP [24]). Therefore, the method described by [23] might not work as well in other settings where stimuli do not include faces.

The idea of multi–brain collaborative decision as proposed by [23] has recently been applied to Brain Computer Interface (BCI) research. For example, in [25] the performance of single and offline collaborative BCI in a task of movement planning have been compared. In the experiment described, through directly extracting information from the posterior parietal cortex and bypassing the motor related procedures, the collaborative BCI system could accelerate a motor response by using an artificial limb. However, this was achieved at the expense of accuracy: even when integrating up to 20 users, this was never above 95% (while performance of a non-BCI single user was 100%, with average response times of 464 ms).

In [26] an online collaborative BCI for detecting the onset of a visual stimulus presented on a black background was proposed. The presentation of the stimuli produced visually evoked potentials that a collaborative BCI was able to detect more accurately than a single-user BCI. Decisions could be made very rapidly (e.g., at 120 ms from stimulus onset) compared with participants' behavioural responses (response time was 332 ms on average), but with substantially lower accuracy (approximately 85% vs virtually 100% for behavioural responses). Also, in the study there was no decision but only detection of one type of event. A similar methodology was adopted in [27], where as in [23] participants were asked to discriminate between images of faces and images of cars presented for 16.7 ms. However, unlike [23], here the task was changed into a Go/NoGo task requiring participants to respond only when faces appeared. This task was only performed in the offline collection of data to be used for training a Support Vector Machine classifier, while during online tests participants were not required to give any response. However, in the latter participants were given immediate feedback showing both the correct and the actual BCI response. In the study, the online performance of a collaborative BCI involving six individuals (78% accuracy) was superior to that of a single-user BCI (65% accuracy), but both were significantly worse than average behavioural performance (92% accuracy). Decisions, however, could be made within 360 ms, which is approximately 50 ms faster than the average behavioural response time. (It is difficult to determine what influence the feedback had on participants' performance, but we note that the use of feedback limits the applicability of the technique only to decision-making studies where the correct outcome is known beforehand.)

Multi-brain aggregation not only can facilitate rapid analysis of the environment and decision making, but can also assess characteristics such as group emotions, as shown in [28]. There, an experiment was described in which a group's emotional index was obtained by aggregating EEG and electromyographic signals from two individuals who were observing emotion-triggering images.

The studies described in this section show that there can be an advantage, over single user performance, when brain activity of a group of individuals is integrated. Also, the larger the group the better the overall performance (groups of up to 20 people were tested in both [23] and [25]). However, while the collaborative BCIs described above can make faster decisions compared to behavioural ones or single-users BCIs, higher accuracy is never achieved (only in [27] performance of the collaborative BCI was more accurate than the single-BCI's performance).

Very recently [29] we have also started studying the potential of a collaborative approach to BCI. In particular, we developed collaborative versions of our analogue BCIs for real-time 2–D pointer control developed in previous work [30]–[32] where the pointer movement is controlled via the integration of the brain activity of two users. The analysis of performance with three participants indicated that our best collaborative BCI produces trajectories that are statistically significantly superior to those obtained in the single BCI user case.

### Contributions of the Present Study

In the present study we examine the possibility of using neural and behavioural features to improve the accuracy of group decisions in a visual-matching task, where images were presented to observers in taxing perceptual conditions (namely, high perceptual load and high speed of stimulus presentation). As previously discussed, in these cases human perception may not only be incomplete but also incorrect or, at the very least, imprecise. By integrating the neural activity and behavioural responses from multiple observers we hoped to achieve more accurate evaluations of such images. (We reported on preliminary results of this exploration in a conference paper [33], where, however, we used fewer participants, a completely different set of features and less powerful prediction models than those reported in this work. These resulted in much weaker neural correlates of decision confidence and generally poorer performance than those obtained here.)

BCI has hitherto implied the use of brain signals from a single user, but, as seen in the previous section, the technology also gives us access to data pertaining to various cognitive processes which have only recently begun to be investigated in multi-user scenarios. Our research departs from previous work in two important respects.

A first distinguishing feature of the work reported in this article is that here we focus on combining BCI technology with human behavioural responses, in order to achieve more accurate decisions than those obtained by a group of individuals making decisions by a traditional majority rule. Previous work on collaborative BCI, instead, has focused on achieving faster-than-human performance. However, this has been done either at the cost of a significant reduction of accuracy compared to a single BCI user, or at the expense of using a large group of BCI users to achieve the performance of a single non-BCI user (e.g., groups of 7 BCI users were required to achieve the same accuracy of one non-BCI user in [23]).

A second distinguishing feature of our work is that here we have striven to derive neural correlates that can be predictive of the certainty with which decisions are taken. We did not want neural correlates associated with the particular choice of stimuli (e.g., face processing is known to produce distinctive ERPs), a particular task (e.g., Go/NoGo tasks produce very different ERPs in the Go condition, where a motor response is required, than those in the NoGo condition, where no response is provided) or a particular way of giving behavioural responses (e.g., left– and right–hand responses activate motor areas in opposite cerebral hemispheres) as has been done in most of previous work.

The article is organised as follows. In the “Methodology” section we describe the method used in the study, including the novel neural and neural-behavioural features we have defined to capture user certainty and our group decisions strategies based on those features. In the “Results” section, we provide a statistical analysis of these features, we study group decisions based on our decision strategies, relating them to both single-user and group decisions based on the traditional majority rule, and we look at the ERPs produced for different levels of certainty. We also show that only using the fastest respondents in a group to make group decisions may lead to decisions that are both more accurate and faster than those of both single non-BCI users and BCI-assisted whole groups. Finally, we conclude the article with some conclusions on our findings and an indication of possible future work.

## Methodology

Our collaborative BCI involves the combination of three features: (a) the neural features extracted from the EEG signals of each group member (*nf*), (b) the decisions made by each member and (c) the response time (*RT*). As indicated above, response times are indicators of confidence (longer decision times being normally associated with lower confidence). Also, our neural features were specifically designed to represent confidence. Using these features, we applied three different methods to weigh each member's decisions before algorithmically combining them and achieve more accurate group decisions.

To test our ideas in a suitably constrained environment, we used a particularly simple set of visual stimuli, which, however, were presented very briefly thereby making the matching task particularly arduous.

### Participants

We gathered data from 10 participants with normal or corrected-to-normal vision (average age 30.6, SD 9.5; 6 female, 8 right handed), who gave written informed consent to take part in the experiment.

The research was part of a project entitled “Global – engagement with NASA JPL and ESA in Robotics, Brain Computer Interfaces, and Secure Adaptive Systems for Space Applications – RoBoSAS” funded by the UK Engineering and Physical Sciences Research Council (project reference EP/K004638/1) which received ethical approval on the 30th of May 2012 by the Research Director of the School of Computer Science and Electronic Engineering of the University of Essex on behalf of the university's Faculty Ethics Committee.

### Stimuli and Tasks

**Figure 1:**
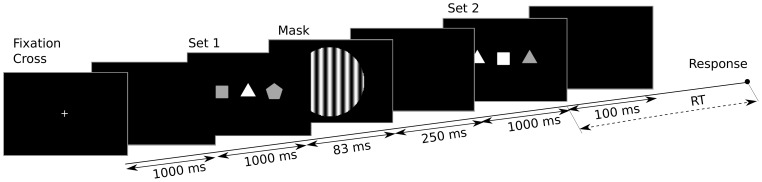
Stimulus sequence used in our experiment. In each trial, a fixation cross was displayed for 1000(a first stimulus composed by three shapes) was presented for 83 ms, followed by a mask (for 250 ms), a black screen (for 1000 ms) and then Set 2 (a second stimulus structurally similar to the first) for 100 ms. The response time, RT, was computed from the onset of Set 2.

Participants underwent a sequence of 8 blocks of trials, each block containing 28 trials, for a total of 224 trials. Each trial (see Figure 1) started with the presentation of a fixation cross in the middle of the screen for 1 second. This time allowed participants to get ready for the presentation of the stimuli and allowed EEG signals to get back to baseline after the response from previous trials. Then observers were presented with a sequence of two displays, each showing a set of shapes. The first set (Set 1) was presented for 83 ms (5 frames of a 60 Hz screen) and was immediately followed by a mask for 250 ms. The mask was a vertical sinusoidal grating with a period of 1 degree subtending approximately 8 degrees. After a delay of 1 second, the second set of stimuli (Set 2) was shown for 100 ms. Following this, observers had to decide, as quickly as possible, whether or not the two sets were identical. Responses were given with the two mouse buttons (left for “identical”, right for “different”), controlled with the right hand, and response times (RTs, expressed in seconds) were recorded (more on this later). Each set consisted of three shapes (subtending approximately 1.5 degrees and being approximately 1.8 degrees apart), which could be any combination of a triangle, square and pentagon (see Sets 1 and 2 in Figure 1). Note that the same shape was allowed to be present multiple times within a set. Each shape was coloured either in pure white (corresponding to normalised RGB (1,1,1)) or light grey (RGB (0.65,0.65,0.65)). Shapes were presented on a black background.

We should note that the right hand was the non-preferred hand for the 2 left-handed participants out of the 10 in our study. While there are typically RT differences when participants use their non-preferred hand over the preferred one, such differences are very small [34] and whether the preferred hand is faster or slower than the non-preferred one depends on the task (e.g., see [35]). So, it is unlikely that this affected in any significant way our results.

With two shades of grey and three possible shapes for each of the three elements in each set, there were a total of (2×3)^3^ = 216 different possibilities for each set, leading to a 216^2^ = 46,656 possible set combinations. Since each element of the three stimuli in a set has two features (grey level and shape), we classified each set pair based on the number of matching features the two sets in the pair shared, a number that we called *degree of match* (DoM). If all three stimuli of Set 1 differ in both shape and grey level from the three stimuli in Set 2, we have a DoM of 0; if one element shares a feature (e.g., the same shape), that is a DoM of 1; etc. So, DoM ranges from 0 to 6 (6 corresponding to a perfect match between Set 1 and Set 2).

Note that a feature was “shared” only when it was in the same position in the two sets. Therefore, if, for example, Set 1 showed a triangle in the first position, while Set 2 showed a triangle in the second or third position, but not in the first position, that was not a shared feature.

The combination of the shapes in Set 1 and their grey levels were randomly selected. However, we found that randomly selecting even the features of Set 2 would produce a disproportionate number of sets which had an intermediate DoM, thereby under-representing the cases where a decision is particularly difficult and also the “identical” condition. So, we imposed a constraint that while stimuli would be random, there should be equal proportions of each DoM category in each block. Once randomly generated, the sequences of sets were stored, so that identical sequences were used for all participants.

There are two reasons for this. Firstly, this ensures that all participants underwent exactly the same experiment, which should increase repeatability and reproducibility. Secondly, as we will explain later, this allowed us to test offline the benefits of combining the decisions of multiple users when presented with identical displays.

The experimental blocks were preceded by a session of practice to allow participants to familiarise with the task and the stimuli. Participants were seated comfortably at about 80 cm from an LCD screen. EEG data were collected from 64 electrode sites using a BioSemi ActiveTwo EEG system.

Briefing, preparation of participants (including checking and correcting the impedances of the electrodes used for EEG recording) and task familiarisation took approximately 30 minutes.

### Data Acquisition and Transformation

#### Response Time Measurement

To measure response times we used button clicks on an ordinary USB mouse. The USB polling rate was 125 Hz. So, the maximum hardware jitter on the RT measurement was 8 ms (the sampling period). In the software controlling the presentation of the stimulus and the synchronisation with the ActiveTwo EEG system, mouse click events were captured in a while loop the body of which only contained a 5 ms sleep. This adds a jitter of at most 5 ms. Finally, we marked the EEG status channel of the ActiveTwo device with the event, which had a further maximum jitter of 1 ms. So, the total maximum jitter on RT was 14 ms. Response times in our experiment were typically around 700 ms, but the shortest recorded RT across all trials and participants was 251 ms. So, in the worst possible case the relative error introduced by jitter in our RT measurements could have been 7.2%, but on average we expect jitter to have affected measurements by less than 2%.

#### EEG Signal Acquisition and ERPs

The EEG channels were referenced to the mean of the electrodes placed on each earlobe. The data were initially sampled at 2048 Hz and were then band-pass filtered between 0.15 and 40 Hz with a 14677-tap FIR filter obtained by convolving a low-pass filter with a high-pass filter both designed with the window method. A form of correction for eye-blinks and other ocular movements was performed by applying the standard subtraction algorithm based on correlations [36] to the average of the differences between channels Fp1 and F1 and channels Fp2 and F2. The data were then low-pass filtered with an optimal 820-tap FIR filter designed with the Remez exchange algorithm [37] with a pass band of 0–6 Hz and a stop band of 8–1024 Hz. The data were finally down-sampled to a final sampling rate of 16 Hz.

The EEG data were segmented into epochs for the purpose of extracting our neural feature. Normally in ERP analyses epochs start in synchrony with the presentation of the stimulus (they are “stimulus-locked”) and last for a certain time. However, here we were also interested in the neural processing that immediately precede and follows a participant's response. This is best captured by using a “response locked” approach, where epochs start a pre-fixed amount of time before the user's response. So, we decided to look at two epochs of data: one lasting 1500 ms and starting on the onset of Set 2 (we wanted to capture the neural signals that immediately follow this stimulus since they might reflect the degree of accuracy, or the level of attention, this stimulus was perceived with) and one lasting also 1500 ms and starting 1000 ms before the response (i.e., the epoch ends 500 ms after the response time). However, the stimulus-locked component was not used in the final system as the information about decision confidence is more evident in the response-locked epochs, as we will see in the “ERP Analysis” section. Thus, at a sampling rate of 16 Hz epochs encompassed 24 time samples for each of the 64 EEG channels used.

#### Space-Time Feature Extraction

Various linear transformations and basis changes on EEG signals have been proposed in the literature to combine different channels and to extract meaningful components. Principal Component Analysis (PCA) has been used as a tool for the analysis of EEG and ERPs since the mid sixties [38]–[40]. PCA is based on the idea that the data are a linear combination of “principal components” which need to be identified. PCA components are orthogonal and they maximally account for the variance present in the data. Because of this, it is often possible to accurately represent the original data with a small set of components. Spatial PCA is used in ERP analysis to find components that represent the covariance in the measurements taken at different electrodes, typically measured over multiple epochs. These components are obtained by extracting the eigenvalues and eigenvectors of the covariance matrix. More recently Independent Component Analysis (ICA) [41] has seen considerable popularity in EEG and ERP analysis [42]–[44]. If a set of signals is the result of linearly superimposing statistically independent sources, ICA can decompose the signals into their primitive sources or “independent components”. When ICA is applied to the signals recorded at different electrodes on the scalp, it can separate important sources of EEG and ERP variability. This can then be exploited, for example, to remove artifacts. In [45] we introduced an alternative representation for EEG signals based on a set of functions, which we called *eigenbrains*, that are particularly suitable to represent the large-scale dynamics associated with ERPs. The method has some similarity with PCA in that eigenbrains are the eigenvectors of a matrix. However, unlike for PCA, this does not use the covariance matrix, but a matrix that represents an approximate model of the brain as a collection of coupled harmonic oscillators.

In this work, we decided to adopt a spatio-temporal PCA (as this is an established and well-tested technique). In this version of PCA we simply treat the channels and the time steps in an epoch as separate stochastic variables. Therefore, with 64 channels and 24 time steps per epoch we obtained a 1,536×1,536 covariance matrix, the eigenvectors of which represent a new set of basis vectors for representing ERPs. The values of the corresponding 1,536 features are simply obtained by performing the dot product between each basis vector and the voltage values in an epoch.

#### Feature Selection

Naturally, it is always difficult to deal with a highly dimensional feature space such as the one defined by our spatio-temporal PCA and, so, some form of feature selection is required. Fortunately, with PCA, the basis functions can be ordered in terms of representational importance on the basis of the magnitude of the corresponding eigenvalues, as these represent the amount of variability/variance in the data represented by each component. Considering only the *n* basis functions corresponding to the *n* largest eigenvalues is, therefore, a simple form of feature selection. As this has proven effective in many cases, in this work we adopted this strategy and selected the first 24 principal components as features. This corresponds to a 1 to 64 reduction from the original 1,536 features.

Note, however, that since PCA aims at capturing the features of the EEG signals irrespective of the task at hand, the feature selection strategy adopted here may not necessarily be optimal for the task at hand. In future work we plan to test more sophisticated forms of feature selection.

### Neural and Behavioural Correlates of Confidence in Decision-making

One of the aims of our study was to identify a neural feature representing the degree of certainty of the decision taken by an observer. However, ground truth information on confidence is not directly available. One can ask a participant to tell his or her degree of confidence in a decision, but it is not clear how objective this measure of confidence would be. So, here we concentrated on trying to find a more objective surrogate of the certainty of a decision. In particular, we tried to characterise the cases where the response given by a user was correct *vs* the cases where the response was incorrect. In a rational observer, we can safely assume that incorrect responses are so because the perceptual processes leading to the decision did not provide all the necessary information to take the correct decision. It thus stands to reason that in these conditions the confidence with which an observer took a decision would be low for most of the “incorrect” trials. On the contrary, the confidence with which an observer took a decision would be higher for most of the “correct” trials.

Of course, when one is not confident as to what he or she has seen, random guessing may be a significant element in the decision. When an observer guesses, it is possible that he or she will give the correct response just by shear luck. So, a fraction of the trials where a correct response is recorded may be characterised by low confidence in the decision. However, if the proportion of correct decisions is sufficiently high (as in our experiments), in the majority of “correct” trials the observer's confidence will be significantly higher than for “incorrect” trials.

Thus, finding predictors of whether the decisions made by a participant will be correct or incorrect would essentially amount to finding predictors of the degree of certainty of the participant in making such decisions.

To perform this task, we used a method based on a form of multivariate linear regression known as Least Angle Regression (LARS) [46]. We should note that LARS is normally used for identifying the coefficients of a linear model while at the same time optimally deciding which variables/features should be included in the model. However, in this work we only used LARS as a regression algorithm with a prefixed number of features. We used LARS as in future research we intend to explore its ability to perform feature selection.

To prepare for LARS, we divided up the PCA-transformed epochs within a training set into those where a correct response was given and those where it wasn't. We associated the desired output +1 to trials resulting in an *incorrect* response and −1 otherwise. We then passed the epochs and their corresponding desired output values to LARS. The model described by LARS had the form 
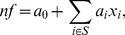
(1)where *nf* is the *neural feature* obtained, 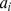 are constant coefficients, 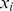 are the features representing an epoch (in the spatio-temporal PCA reference system) and 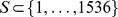 is the subset of the 24 features selected as explained above. We can now use this neural feature to express the certainty of the participant in the response given and weigh its influence in the group's decision – see below. Note that LARS was applied on a participant-by-participant basis.

Another method that we have used to measure the confidence in decisions made by the participants involves the response times *RT* as a *behavioural correlate*. As described in previous sections, slower response times are generally associated with uncertainty in the decision made by observers, while faster response times mean that participants were more confident in their decisions. This information can be used, similarly to the neural feature described above, to weigh the influence of each observer in the group's decision.

### Using Behavioural and Neural Features in Decision-making

As we indicated above, one of our objectives was to combine the behavioural and neural features from multiple users – in conditions of complete absence of communication or any other form of social influence – to see under what conditions their decisions in a perceptual task would be more accurate than those taken by a single observer. To achieve this, we decided to compare the standard majority rule against rules where the confidence of the observers (as assessed by the RT feature, the nf neural feature or their combination – see below) is used to weigh their decisions.

In the case of majority, all observers' decisions (either a “yes” or a “no”) counts the same. The final decision is based on straight majority for teams with an odd number of members and majority followed by the flipping of an unbiased coin in the case of ties for teams with an even number of members. Of course, to reduce the noise in our performance estimates we didn't actually flip a coin. Instead, we used the expected value of the outcome of the decision. That is, when counting the number of correct decisions we added 0.5 to the count for every decision where there wasn't a majority since such decision would turn up to be correct in exactly 50% of the cases.

For the other methods, the decision made by each observer is weighed according to the information about certainty given by the features used (*RT*, *nf* or a combination of them). For each participant, *i*, within a group, a decision weight, 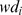, is computed (on a trial-by-trial basis). Then, we take the following joint decision: 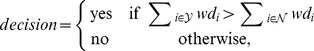
(2)where 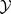 and 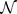 represent the sets of all observers who decided “yes” and “no”, respectively.

The weights 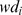 for *RT*-based and *nf*-based methods were set by transforming the corresponding feature through the following *negative exponential weighting function*: 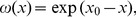
(3)where 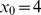 for the *RT*-based method and 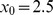 for the *nf*-based method. Thus, when *RT* was used as a measure of confidence, we set 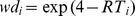, where 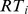 is the response time for observer *i* in a particular decision. Similarly, when *nf* was used to express certainty, we set 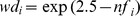, where 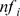 is the neural feature obtained from the LARS model in Equation (1) for observer *i* corresponding to a particular decision.

**Figure 2:**
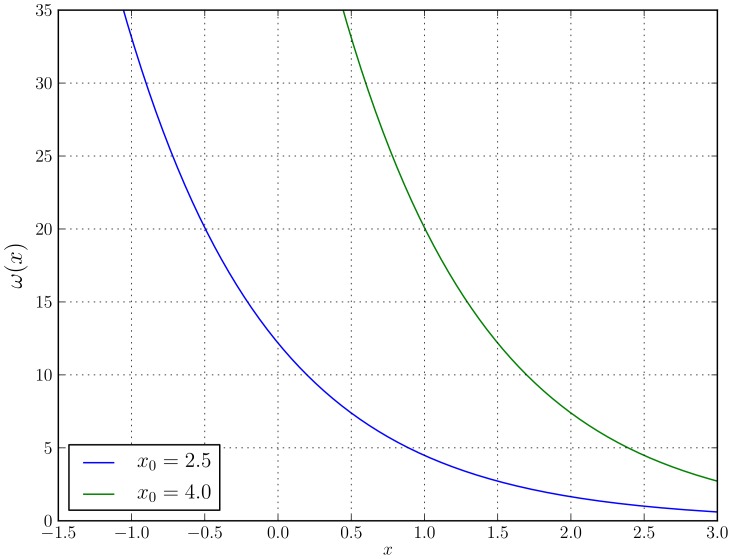
Plots of the negative exponential weighting function 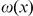 adopted in our studies (see Equation (3)) to transform neural and behavioural correlates of confidence into decision weights. The green line represents the weighting function used for response times, while the blue line represent the function used for the *nf* feature. The shape of these functions allows confident decisions to count more than uncertain ones.

Plots of the weighting function in Equation (3) for the two methods are shown in Figure 2. The key reason for using a negative exponential function is to allow very confident individuals to count substantially more than uncertain or not so confident individuals in the group's decision. This allows neural (*nf*) and behavioural (*RT*) correlates of decision confidence not just to meaningfully break ties but also to swing the joint decision in favour of a confident minority when the majority is sufficiently uncertain. This function is also desirable as it is always positive, avoiding negative weights which would imply changing “yes” decisions into “no” decisions or *vice versa*. The choice of 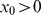 was to ensure there was reasonable variation in weights for both *RT*-based and *nf*-based methods (as variation is a necessary condition to do better than the majority rule). The particular choices of 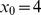 when *RT* is used as a measure of confidence and 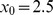 when *nf* is used were determined by the desire to make the magnitude of the 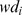 produced by the two methods roughly comparable.

We ask LARS to produce an output of -1 for correct decisions and +1 for incorrect ones. Since participants typically make 10–15% incorrect decisions, the average value for *nf* is between −0.7 and −0.8. On the contrary, *RT* is always positive, and typically around 0.7 seconds. So, by shifting the 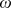 functions used in the two methods by values of 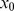 differing by 1.5, we are reasonably sure that weights for these methods will be in approximately the same range.

We also defined a third method (which we will call “neuro-behavioural” or “*RTnf*-based” for reasons that will become immediately apparent) for setting the decision weight 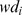 for the contribution of a participant in a group's decision using a linear combination of the weights obtained with the *RT*-based and *nf*-based methods. More specifically, we set 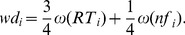
(4)where 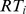 is the response time for observer *i* in a particular decision, 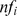 is the neural feature obtained from the LARS model in Equation (1), and 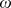 is the weighting function described in Equation (3).

The choice of the coefficients 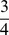 and 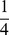 was simply guided by our experience with BCIs. BCIs tend to be relatively unreliable in single-trial classification tasks. So, as our system requires trial-by-trial decisions, by giving more influence to the confidence weight inferred from *RT* we attempted to compensate for the higher noise expected in *nf*. By combining these two methods we hoped to obtain a more robust confidence measurement which would then result in better decisions.

### Learning Neural Correlates of Confidence

While in the *RT*-based method no machine learning or adaptation of the model takes place, this is required when *nf* is used to measure the confidence in the decision made. Our application of LARS relies on the extraction of information from the ERP data via PCA. Then LARS automatically selects the coefficients for each component. Since this is a form of machine learning, we must ensure that our results are not affected by over-fitting.

Ordinarily, validation of methods such as ours requires splitting the available data into a training and a test set. Because the data sets one can acquire in electrophysiology, neural engineering and BCI studies are always relatively small compared to other domains, we adopted, as is customary, a *k-fold cross-validation* approach. In each fold we had a training set of 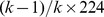 trials and a test set of 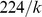 trials. We computed the PCAs and the linear regression coefficients of LARS only using the training set. We then reused these same values to estimate our features *RT* and *nf* and the corresponding weights 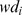 as well as the weights associated to the *RTnf*-based method for the trials in the test set. In order to ensure all folds had the same number of samples, as the number of trials (224) is divisible by 7 and by powers of 2 up to 2^5^, we used *k* = 2, 4, 7, 8, 14, 16, 28, 32, 56, 112 and 224 (leave-one-out strategy) in our experiments, although we will mainly report results for *k* = 16 (performance varied very little with *k* as we will illustrate below).

### Group Decisions based on Fastest Responders

As we discussed in the “Introduction” section, it is known that response times are influenced by, and thus can reveal, the confidence in a decision [22]. Typically, faster responses are correct more often than slower responses. We decided to try to exploit this effect within the context of group decisions. So, in addition to the group decision methods discussed above, we also tested the idea of making group decisions by only considering the decisions of the faster responders in a group, as will be described in detail in the “Results” section.

## Results

### Individual Decisions

To start our analysis, we looked at the differences in performance shown by our participants when performing the task in isolation and without any manipulation (weighting) of their decisions.

**Figure 3:**
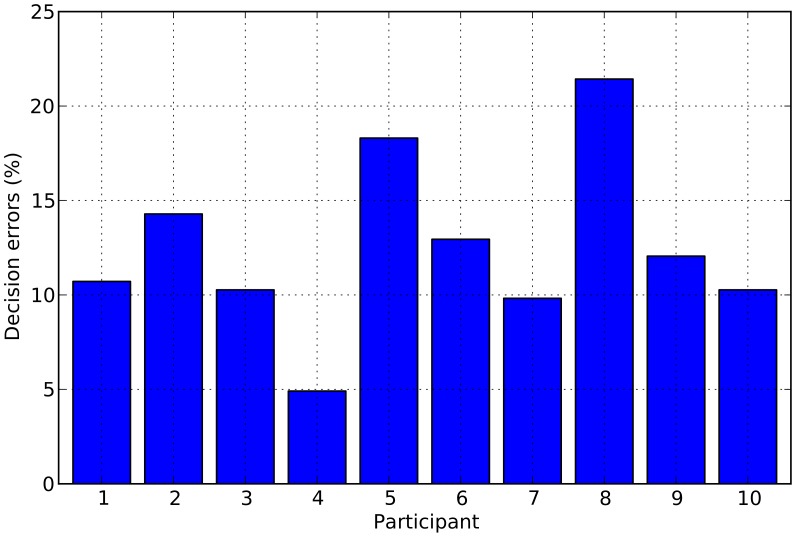
Percentage of erroneous decisions made by each participant in the 224 trials of our experiment. Error rates ranged form 5% to over 20% with an average error rate of 12.5%.

The average error rate in the visual matching task used in our experiment across all participants was 12.5%. However, as one might expect, participants showed radically different individual levels of performance as illustrated in Figure 3, with error rates ranging from just below 5% to over 20%. Interestingly, if we look at the subset of trials where matching pairs of stimuli were presented, we see that participants gave incorrect decisions in only 0 or 1 out of the 28 matching pairs, thereby showing a very high sensitivity to identical sets. The bulk of the errors, instead, were due to participants having decided to classify as “matching” stimuli that actually did not match.

This pattern was common to all our participants except one that, for unknown reasons, showed a much larger number of missed matching pairs and overall gave responses that were hardly distinguishable from random. So, this participants data were discarded and replaced with data from a new participant.

### Behavioural, Neural and Neuro-behavioural Features

Let us now turn our attention to our neural and behavioural correlates of decision confidence.

To investigate the relationship between correct/incorrect responses and the confidence with which decisions were taken, we studied the distributions of the decision weights 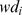 associated with *RT* and the neural feature *nf*, as well as the those based on neuro-behavioural feature *RTnf* obtained as indicated in Equation (4).

**Figure 4:**
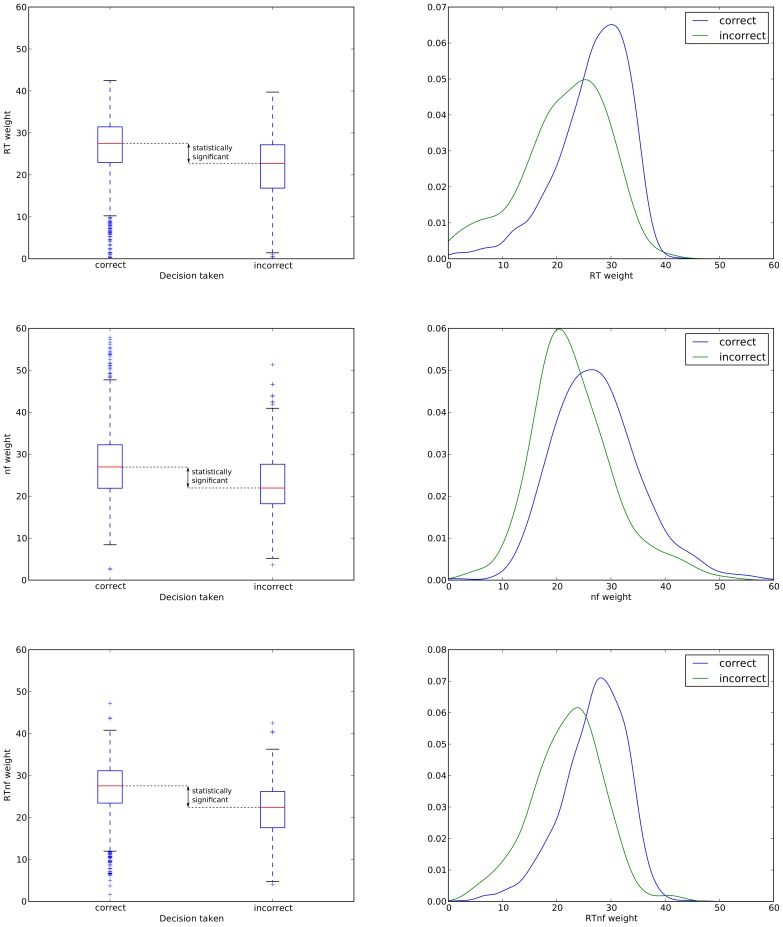
Box plots representing the distributions of the weights for different features and for different decisions (left) and corresponding probability density functions estimated via Gaussian kernel density estimation. As indicated graphically in the plots on the left and as discussed in the text, the differences between correct and incorrect decision weights are highly statistically significant for all methods.

We started by binning the data (obtained via cross-validation) on the basis of whether a decision made in a trial by an observer was correct or incorrect. Table 1 reports the medians of the decision weights associated to the behavioural feature *RT* and the neural feature *nf*, and the neuro-behavioural mixing of the two, *RTnf*, for correct and incorrect trials. The corresponding box plots and density functions (obtained via a kernel-based estimator) are shown in Figure 4. As one can see from these, the medians of the decision weights are significantly lower for the incorrect decisions than for the correct ones for all the features used. These differences resulted to be highly statistically significant when we applied both the Kruskal-Wallis test (a one-way, non-parametric, analysis-of-variance test roughly equivalent to the parametric ANOVA test) and the Wilcoxon rank sum test to the data. In all comparisons and for both tests 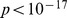 (with statistics *H*>77.6 and *W*>363,600) in all cases. These tests indicate that trials where the decision weights are characterised by lower values were also those where decisions were more difficult (and were, therefore, taken with a high level of uncertainty) than those characterised by higher weights.

**Table 1:** Medians (across all participants) of the decision weights 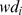 associated to behavioural, neural and neuro-behavioural methods presented in this paper, as a function of whether the user's response was correct or incorrect.

	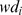		
*Decision*	*RT*	*nf*	*RTnf*
correct	27.514	26.967	27.543
incorrect	22.721	21.943	22.412

We should note that the use of the above-mentioned non-parametric tests was required as the distributions of decision weights (see Figure 4(right)) are clearly non-Gaussian. We used the version of the Wilcoxon test included in the R package exactRankTests which computes the exact test even in the presence of ties. Sample sizes, which in non-parametric tests play a role similar to the degrees of freedom for parametric ones, were 1960 for the “correct” class and 280 for the “incorrect” class.

We then binned the data on the basis of the degree of match of the stimuli presented in each trial (as an indicator of the objective difficulty of the task of discriminating them). Table 2 reports the medians (across all participants) of the decision weights 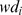 associated to different features as a function of the DoM of the stimuli used in a trial. Overall, as we hypothesised, stimuli configurations characterised by higher DoM, which are thus objectively harder to decide upon, are associated with lower 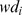, suggesting that our neural and behavioural features do indeed capture the confidence of decisions.

**Table 2:** Medians (across all participants) of the decision weights 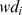 associated to behavioural, neural and neuro-behavioural methods, as a function of the degree of match, DoM, of the pair of stimuli used in a trial.

	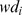		
*DoM*	*RT*	*nf*	*RTnf*
0	29.410	28.286	29.236
1	29.396	27.881	28.836
2	28.673	27.939	28.936
3	27.041	26.701	27.151
4	26.726	25.224	26.686
5	24.399	24.923	24.045
6	22.904	22.591	23.030

The DoM is the number of identical features the two stimuli in a pair have. Since each stimulus contains 6 features (the shape and colour of three polygons), in our experiments a pair including two *identical stimuli* has DoM of 6.

### Group Decisions

We compared the performance of single observer decisions with group decisions within groups of increasing size. All possible memberships of the groups were tested. There are 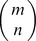 distinct groups of size *n* constructed from a population of *m* observers. Since we had *m* = 10 observers, we had 10 “groups” of sizes 1, 45 groups of 2 observers, 120 groups of 3, 210 groups of 4, and so on.

For each *group* we computed the number of errors made by the group when using the four different methods of making decisions studied in the paper (i.e., based on majority rule and our three features *RT*, *nf* and *RTnf*). For each *group size* we then computed the mean number of errors made with each method.

**Figure 5:**
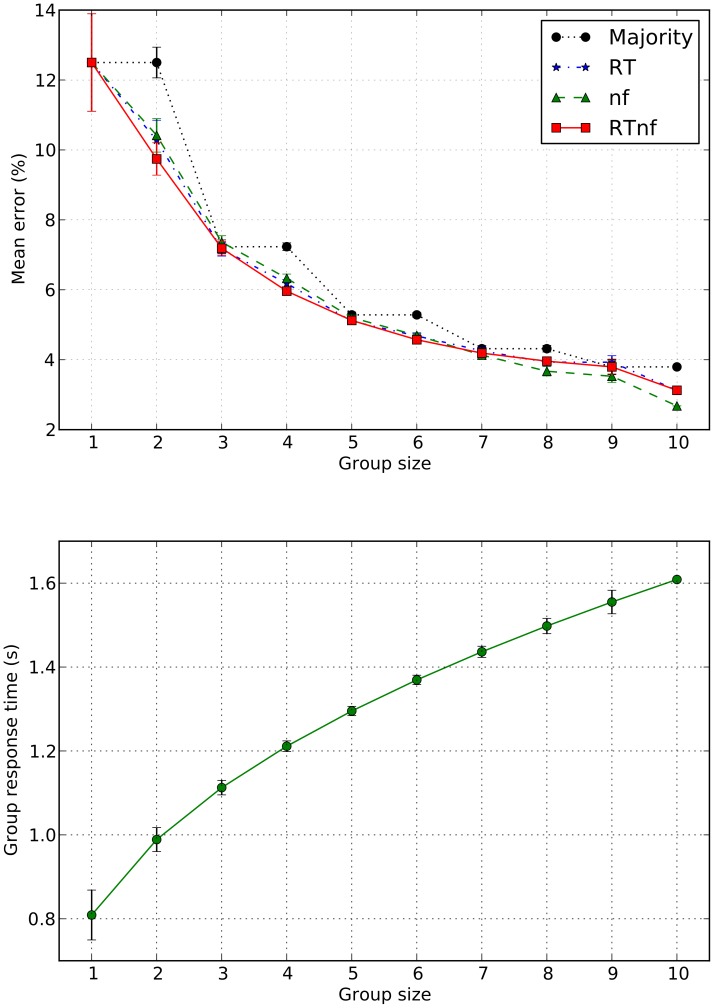
Average percentage of errors *vs* group size for the four methods for group decisions tested in this paper (top) and average time required for groups of each size to make a decision (bottom). The plots also show error-bars representing the standard error of the mean for each group size, except for groups of size 10 for which this cannot be computed as only one measurement is available. Statistical comparisons for the error rates shown in the top plot are detailed in Tables 4 and 5 and are represented graphically in Figure 7.

In Figure 5(top), we report the average percentage of errors as a function of group size for the four methods for group decisions tested in the paper. The data are also reported in numerical form in Table 3. As one can see, in all methods studied except that using majority rule for groups of size 2, group decisions were superior to the decisions of single observers (we will look at the statistical significance of this finding shortly), suggesting that integration of perceptual information across non-communicating observers is possible and beneficial. Also, we see that the straight majority is generally outperformed by the other three methods. This is particularly evident with groups having an even number of members where the coin-tossing required by majority rule in the presence of ties implies that performance is the same as that of groups with one fewer member. The data also show that of the three other methods, the *RTnf*-based method appears to be the most consistent, being best or second best in 9 out of 10 cases. The data also suggest that with large group sizes (from 7 upward) the performance of majority starts saturating possibly to a worse asymptote than the performance of the methods based on confidence correlates.

**Table 3:** Average error rates (%) *vs* group size for the four methods for group decisions tested in the paper.

*Group Size*	*Majority*	*RT*	*nf*	*RTnf*
1	**12.50**	**12.50**	**12.50**	**12.50**
2	12.50	*10. 27*	10.41	9.74
3	7.23	**7.16**	7.36	*7. 18*
4	7.23	*6.18*	6.32	**5.96**
5	5.28	**5.10**	5.20	*5.12*
6	5.28	*4.67*	4.69	**4.57**
7	4.31	4.25	**4.13**	*4.18*
8	4.31	*3.92*	**3.67**	3.95
9	3.79	3.92	**3.52**	*3.79*
10	3.79	*3.12*	**2.67**	*3.12*

The minimum error rate for each group size is shown in bold face, the second best is in italics.

It is also interesting to note that while performance of the *nf*-based method appears to be inferior to *RT*-based and *RTnf*-based methods for groups of sizes 2 to 6, it is the best method for groups of 7, 8, 9 and 10 members. This suggests that our choice of coefficients in Equation (4), while making *RTnf* a generally good all-rounder, may have been suboptimal for the larger groups. We will explore this issue in future research.

We will look at the statistical significance of these observations later in this section. However, before doing this we want to make two observations.

Firstly, let us focus on decision times. In Figure 5(bottom) we report the average time required by groups of each size to make a decision after the presentation of the second stimulus set. Since all groups members must have made their decision before the group can give a response, a group's response time is the maximum response time across group members. Unsurprisingly, the higher accuracy shown by bigger groups in Figure 5(top) comes at the cost of an increase group response time. In most cases it is unlikely that waiting an extra few hundreds milliseconds would be a problem. However, in the next section we show how the problem can be bypassed.

**Figure 6:**
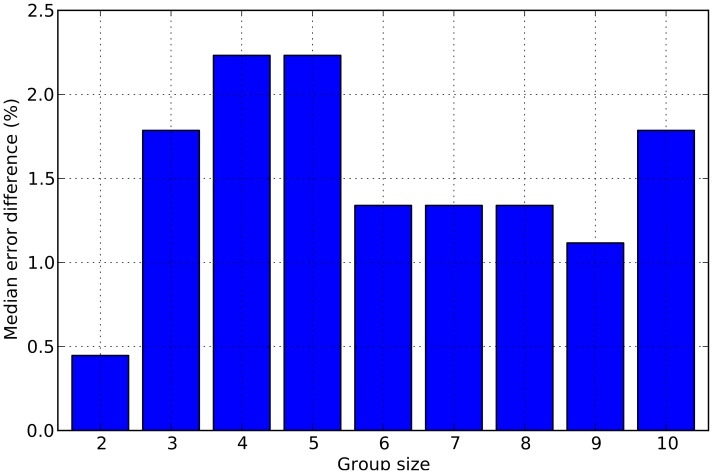
Medians of the differences in error rates between group decisions made with *RTnf* and decisions taken by the best performer in each group. Positive values indicate the extent to which groups were *better* than their best performers.

Secondly, the improvement in performance seen in groups of increasing size might simply be due to the increased likelihood of inclusion of the top-performing participants in the larger groups. For instance, our top performer, participant 4, will only be included in 20% of the groups of size 2, in 50% the groups of size 5 and 90% of the groups of size 9. It is possible that the presence of that participant in a group would be sufficient to drive the error rate of the groups downward significantly. In principle, it might be the case that groups don't do better than their best performer. Of course, we know that this is not the case, at least for groups of size 6 or above, simply because the group error rates are *below* the error rate of our top participant. However, to investigate this issue more thoroughly, for each group of a given size, we have compared the performance of the group obtained by our *RTnf*-based method to that of its best individual performer. Figure 6 reports the median difference in error rates between the two, for each group size. The figure makes it quite clear that group decisions are to a significant extent the result of a process of integration of confidence across participants, and not only the result of top performers driving group errors down.

To test if the observed differences in error rates in Figure 5(top) and Table 3 are statistically significant, we compared the *distributions* of errors made. We started by comparing the error distributions of single observers with the error distributions of groups of increasing size (for the four methods of group decision tested) using the Kruskal-Wallis statistical test. Table 4 reports the *p*-values and statistics returned by the test when comparing the performance of single observers against the performance of groups of different sizes and adopting different decision methods. This shows that for groups of size 2, the *RTnf*-based method is very close to be statistically significantly better than single observers, while for the *RT*- and *nf*-based methods the overlap of the distributions and sample sizes are such that statistical significance is not achieved despite the performance of all methods being on average 2 to 3% better than the single observers' case (as shown in Figure 5). On the contrary, for groups of size from 3 to 9 group decisions are always significantly superior to single observers. Finally, we should note that our group of size 10 is, unsurprisingly, not significantly superior to single observers, even though its performance is superior that of *all* the single observers (see Figure 3), due to it being a sample of just one data point.

**Table 4:** *p*-values and corresponding *H* statistics (in brackets) returned by the Kruskal-Wallis test when comparing the performance of single observers against the performance of groups of different sizes and adopting different decision methods.

*Group Size*	*Majority*	*RT*	*nf*	*RTnf*	*Sample sizes*
2	0.751561 (0.1)	0.088386 (2.9)	0.274314 (1.1)	0.050447 (3.8)	10, 45
3	**0.000094** (15.2)	**0.000080** (15.5)	**0.000077** (15.6)	**0.000070** (15.8)	10, 120
4	**0.000065** (15.9)	**0.000009** (19.7)	**0.000011** (19.3)	**0.000006** (20.5)	10, 210
5	**0.000002** (22.4)	**0.000002** (23.0)	**0.000002** (22.6)	**0.000002** (22.9)	10, 252
6	**0.000003** (21.7)	**0.000001** (24.1)	**0.000001** (24.2)	**0.000001** (24.5)	10, 210
7	**0.000001** (24.9)	**0.000001** (24.9)	**0.000000** (25.6)	**0.000000** (25.5)	10, 120
8	**0.000002** (22.4)	**0.000002** (23.0)	**0.000001** (23.3)	**0.000002** (23.0)	10, 45
9	**0.000174** (14.0)	**0.000172** (14.1)	**0.000146** (14.4)	**0.000146** (14.4)	10, 10
10	0.113024 (2.5)	0.113024 (2.5)	0.113024 (2.5)	0.113024 (2.5)	10, 1

Samples sizes are indicated in the last column of the table. *p*-values below 0.01 are in bold face.

We then compared the error distributions across the group-decision methods *within* each group size. Since errors are paired in each comparison (by the fact that the two methods being compared were applied to exactly the same groups), here we used the one-tailed Wilcoxon signed-rank test. The corresponding *p*-values and statistics are reported in Table 5.

**Table 5:** Statistical comparison of methods for group decisions for different group sizes.

	*Group size*									
*Comparison*	2	3	4	5	6	7	8	9	*Wins*	
Maj. wins over *RT*	1.000000 (863.5)	0.849404 (1454.5)	1.000000 (18417.0)	0.999980 (11030.0)	1.000000 (15137.0)	0.823287 (1679.0)	0.999809 (633.0)	0.312500 (7.5)	0	
Maj. wins over *nf*	1.000000 (930.5)	**0.007901** (1013.5)	1.000000 (17144.5)	0.955750 (9596.5)	1.000000 (16909.5)	0.999936 (2419.0)	1.000000 (868.0)	1.000000 (10.0)	1	
Maj. wins over *RTnf*	1.000000 (939.0)	0.840412 (854.0)	1.000000 (20671.0)	0.999998 (7044.0)	1.000000 (18062.0)	0.996111 (1855.0)	0.999983 (728.5)	0.687500 (5.0)	0	
*RT* wins over Maj.	**0.000000** (82.5)	0.151777 (1101.5)	**0.000000** (1086.0)	**0.000020** (5441.0)	**0.000000** (2068.0)	0.178954 (1324.0)	**0.000199** (147.0)	0.781250 (13.5)	5	
*RT* wins over *nf*	0.203692 (384.5)	**0.009023** (1543.0)	*0.032233* (6621.5)	*0.029350* (8523.5)	0.516544 (8355.5)	0.949481 (2448.0)	0.982161 (536.5)	0.968750 (23.5)	3	
*RT* wins over *RTnf*	0.986913 (496.5)	0.182679 (1158.5)	0.999996 (8525.5)	0.183751 (4996.5)	0.998655 (4787.5)	0.949677 (1188.0)	0.339478 (174.0)	0.781250 (10.5)	0	
*nf* wins over Maj.	**0.000000** (59.5)	0.992283 (1912.5)	**0.000000** (1965.5)	*0.044261* (7239.5)	**0.000000** (2005.5)	**0.000066** (902.0)	**0.000000** (35.0)	0.062500 (0.0)	6	
*nf* wins over *RT*	0.798069 (518.5)	0.991064 (2735.0)	0.967820 (9131.5)	0.970692 (11576.5)	0.483743 (8297.5)	0.050644 (1647.0)	*0.018418* (243.5)	0.062500 (4.5)	1	
*nf* wins over *RTnf*	0.972059 (630.5)	0.998617 (2322.0)	0.999997 (10578.5)	0.958911 (9438.5)	0.963843 (8594.5)	0.146942 (1370.5)	**0.002492** (130.5)	0.062500 (0.0)	1	
*RTnf* wins over Maj.	**0.000000** (7.0)	0.163370 (631.0)	**0.000000** (444.0)	**0.000002** (2826.0)	**0.000000** (1441.0)	**0.003941** (920.0)	**0.000018** (132.5)	0.687500 (5.0)	6	
*RTnf* wins over *RT*	*0.013334* (206.5)	0.818191 (1469.5)	**0.000004** (3720.5)	0.816583 (5881.5)	0.001365 (2593.5)	0.053833 (765.0)	0.675346 (204.0)	0.281250 (4.5)	3	
*RTnf* wins over *nf*	*0.028336* (315.5)	**0.001385** (1081.0)	**0.000003** (4646.5)	*0.041145* (7032.5)	*0.036220* (6283.5)	0.853722 (1789.5)	0.997761 (430.5)	1.000000 (10.0)	5	
*Sample size*	45	120	210	252	210	120	45	10		

The table reports the *p*-values and corresponding *W* statistics (in brackets) returned by the one-tailed Wilcoxon signed-rank test when comparing the performance of single observers against the performance of groups of different sizes and adopting different decision methods. Samples sizes (the number of groups of each size) are indicated in the last row of the table. *p*-values below 0.05 are in italics while values below 0.01 are in bold face. The “Wins” column reports the number of group sizes where *p*-values were below the 0.05 statistical significance level.

**Figure 7:**
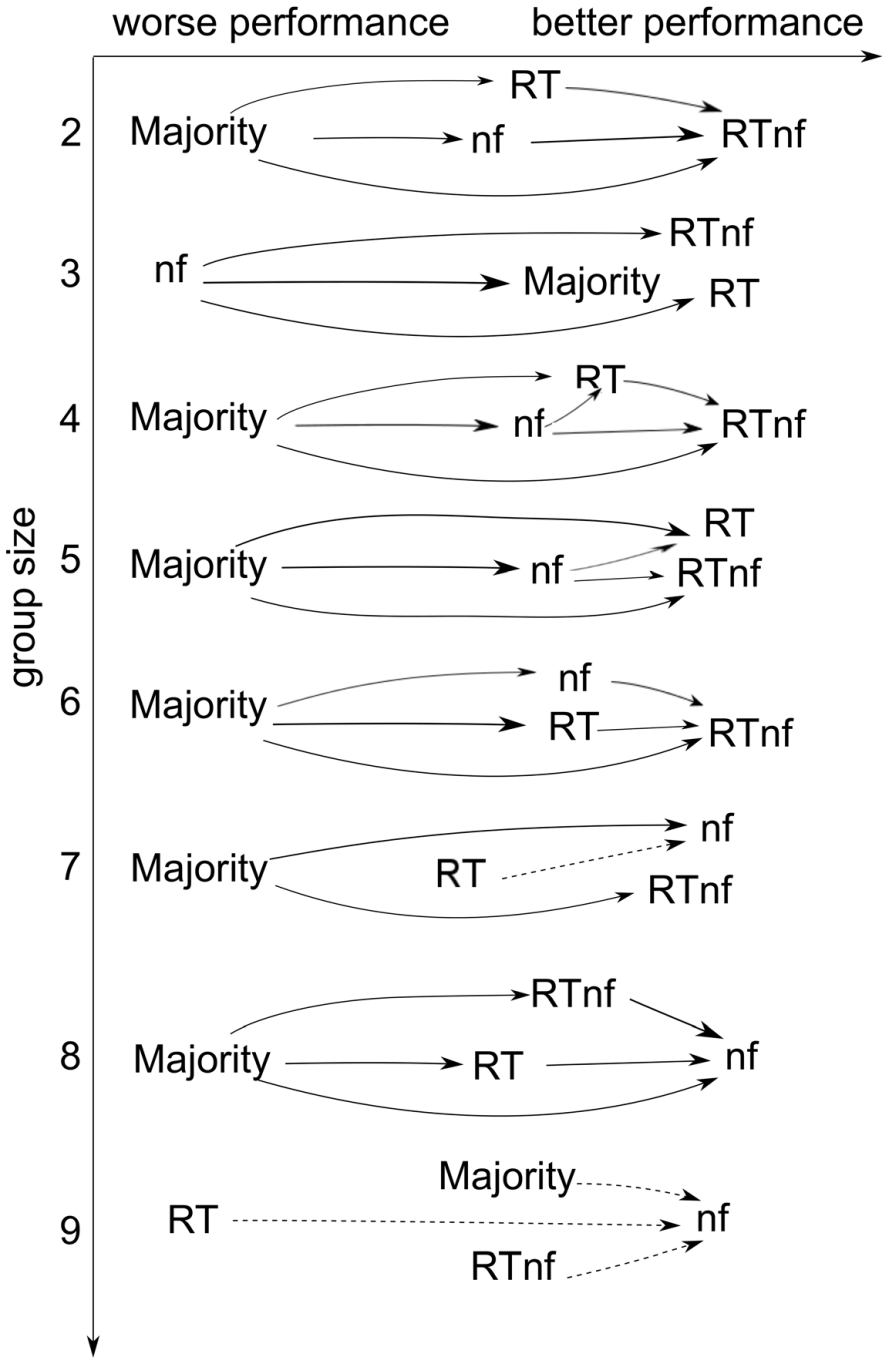
Statistical preference-relation diagram representing the results reported in **Table 5** graphically. For each group size, a one-tailed Wilcoxon signed-rank test was executed, comparing the performance obtained with different decision methods. Solid arrows indicate that the method at the arrow-head is statistically superior to the method at the other end of the arrow (*p*-value lower than 0.01) while dashed arrows indicate near statistical significance (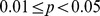).

As expected, we found that several of the small differences shown in Figure 5(top) and Table 3 are not significant. To make it easier to see which differences were significant, we summarise the *p*-values obtained in our tests using the statistical-significance preference-relation diagram shown in Figure 7. Groups of size 1 (all methods performing the same) and 10 (where we only have one such group) are not reported as no difference is statistically significant. For other groups sizes, while at one end of the spectrum we see that majority is statistically almost always the worst method of the four, at the other end we see that the *RTnf*-based method is statistically superior to majority in 6 out of 8 group sizes, is superior to the *RT*-based method in 3 out of 8 group sizes and is superior to the *nf*-based method in 5 out of 8 cases. Both the *nf*-based and *RT*-based methods are also competitive against majority. In particular, *nf* is superior to majority 6 times and almost statistically superior one further time (being inferior to it only for groups of size 3).

Nonetheless, one would probably choose the *RT*-based method if group sizes were small or if there wasn't a need for the slightly better performance afforded by *nf* for larger groups. This is because, of course, using *RT* on its own to measure the confidence does not require the use of a BCI, with its associated and obvious drawbacks in terms of practicality and setup time. However, if top performance is required, the *RTnf*-based method seems to be the overall leader, although had we been able to test larger groups it is likely that the *nf*-based method would have emerged as the leader.

**Figure 8:**
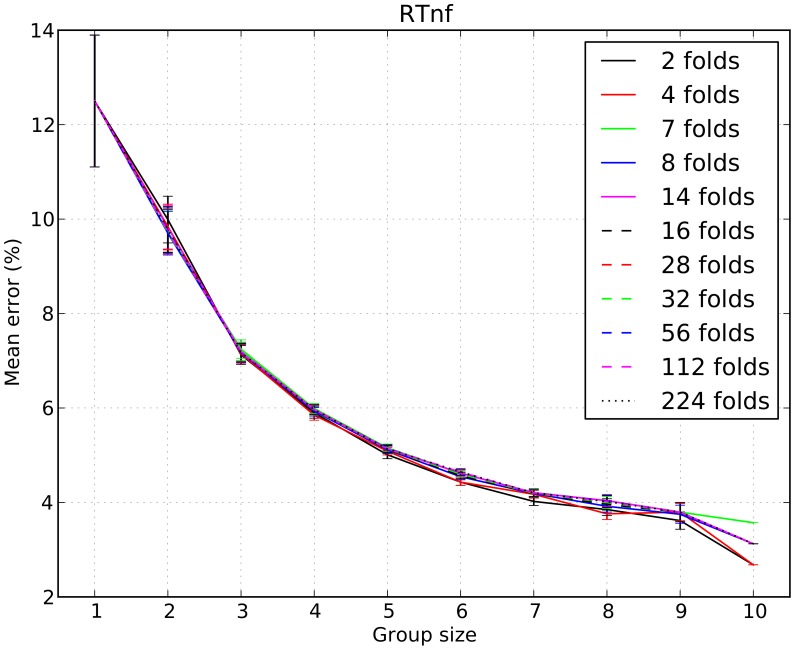
Average percentage of errors *vs* group size and number of cross-validation folds for group decisions made with the *RTnf*-based method. As can be seen from the overlapping error bars (representing the standard error of the mean) and extensive statistical comparisons (see text), performance depends very little on the particular choice of the number of folds used for cross-validation.

We should note that the results obtained by using *nf* and *RTnf* to measure confidence are very little influenced by the number of folds chosen for cross-validation (while, of course, the results of majority and the *RT*-based method are exactly the same for any choice of folds as no learning process takes place in such methods). To illustrate this, in Figure 8 we report the error rates for the *RTnf*-based method as a function of group size and number of folds. The error bars in the plots represent the standard error of the mean. A statistical comparison of the performance obtained with different numbers of folds using the Wilcoxon exact test with Bonferroni correction showed that in only 13.8% of the 550 comparisons required by a full analysis (with 11 cross-validations, there are 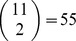 pairwise data-set comparisons for each group size) differences were statistically significant. Also, for most group sizes the differences are very small. This suggests that the case of 16 folds on which we focused in most of the paper is reasonably representative.

### Performance of Fastest Responders

We considered again the relationship between performance and response times. As expected from the literature [22], also in our experiment there is a relationship between the relative speed with which observers give their response and the correctness of the decisions, with faster respondents being on average correct more often than slower respondents (see Figure 4(top right)). Also, as we have seen in Figure 5(bottom) the larger a group the longer the delay in getting the group's response. So, we wondered whether we could improve group response times with relatively little impact to group accuracy if we allowed only the faster responders in a group to influence the group's decision, as described in the “Methods” section. In particular, we considered groups of all sizes and for each size we looked at what level of performance could be achieved by making decisions based on the fastest respondent, the two fastest respondents, the three fastest respondents, and so on, in each trial.

**Figure 9:**
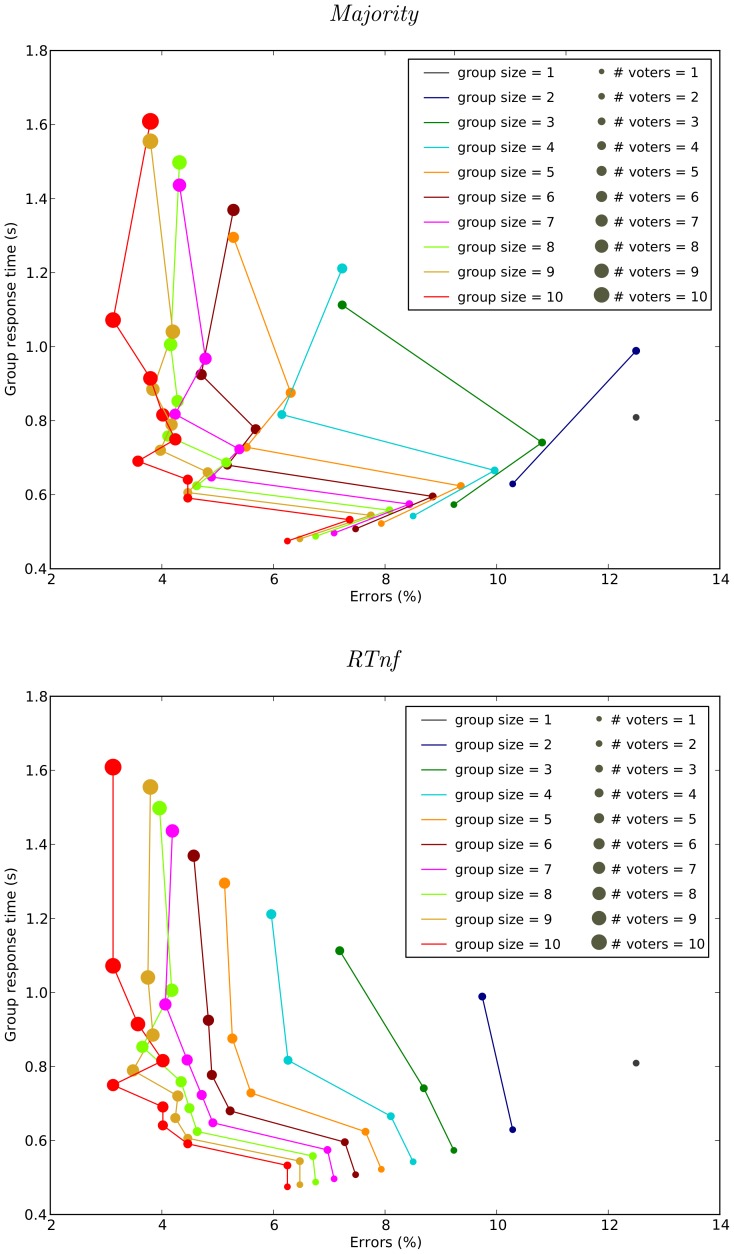
Comparison of the accuracies obtained with different groups sizes and different numbers of voters from within a group against the corresponding response times for the group when using the majority (top) and *RTnf* (bottom) group-decision rules. Each line colour represents a group size. Circles of different diameters represent different numbers of fastest responders (“# voters”) from each group which were allowed to vote.

Figure 9 compares the accuracies obtained with different groups sizes (and different sub-group sizes) with the corresponding response times for a group. More specifically, Figure 9(top) shows a plot of the mean group response time *vs* the mean group error rate for each group size when using the majority method. In the plot, circles of different diameters represent different numbers of fastest responders (“# voters” in the figure) from each group which were allowed to vote. That is, with the exception of the largest circle on each line (which represents the error vs RT trade-off for groups where everyone votes), only the decision of the fastest subgroup were used to determine group decisions. Figure 9(bottom) reports the corresponding results for the *RTnf*-based method. Let us analyse these data.

Firstly, results confirm that the fastest respondents (“# voters = 1”) tend to be the most accurate. On average a single observer has an error rate of 12.5% (see data point for the “group size = 1” case) while selecting the response of the fastest performer in each trial produces an error rate of less than 8% for groups of size 5 or above (irrespective of decision method). Of course, the larger the group considered the shorter the response time of the fastest respondent. So, fastest respondents for groups of sizes 9 take 480 ms on average to make a decision, while the full group takes approximately three times longer (1550 ms).

Secondly, we see that for the majority method there is no gain in using fastest-pair (“# voters = 2”) decisions over fastest-respondent decisions (“# voters = 1”), as the former are both slower and more error-prone than the latter. On the contrary, for the *RTnf*-based method, we see that fastest pairs are almost always more accurate (but slower) than single fastest respondents. For instance, for groups of size 3, single fastest respondents make decisions in 560 ms while pairs take 730 ms. However, while the error rate for fastest respondents is the same (9.2%) for majority and *RTnf*, the error rate for the fastest pair is 10.8% for majority but only 8.6% for the *RTnf*-based method.

Thirdly, we see that when only the fastest triplet of observers (“# voters = 3”) is allowed to make a decision, there is a very marked improvement in accuracy for both majority and the *RTnf*-based method for all group sizes. The benefits of such a scheme are particularly clear for larger groups where the fastest triplet's response is faster compared with the full group response, while the accuracy is significantly better than for pairs or single fastest respondents. For instance, for groups of size 9, the fastest triplet has an error rate of 4.4% and a response time of 610 ms for both majority and the *RTnf*-based method.

Fourthly, for fastest subgroups of four observers (“# voters = 4”) we see a similar situation to that of the fastest pairs. That is, one never gains from using the fastest four observers to make a decision with majority rule, as accuracy is worse than for the three fastest observers and speed is slower. However, with the *RTnf*-based method we see that, for groups of size 4, 5, 6 and 7, the four fastest observers are more accurate (but obviously slower) than any smaller subgroup. This behaviour seems to be present also at larger subgroup sizes.

### ERP Analysis

We used two statistical tests to analyse our ERP data sets. To get an indication of the differences in the statistical distributions of ERPs for correct and incorrect responses in our data-set, we grouped all ERPs (irrespective of the participant they pertained to) into two corresponding sets. We then applied the Kruskal-Wallis test to compare the voltages measured in each channel at each time step in the two data sets. We also performed a two-tailed Wilcoxon signed-rank test for paired samples to compare the mean ERPs obtained on an individual basis.

We should note that for the central-limit theorem, means tend to be distributed according to a normal distribution. So, in principle one could also use a paired-sample *t*-test to perform this comparison. We performed both this test and the Wilcoxon test on our data. Differences in *p*-values were minimal. Here we prefer to report only the results of the statistically weaker Wilcoxon test as this relies on fewer assumptions.

**Figure 10:**
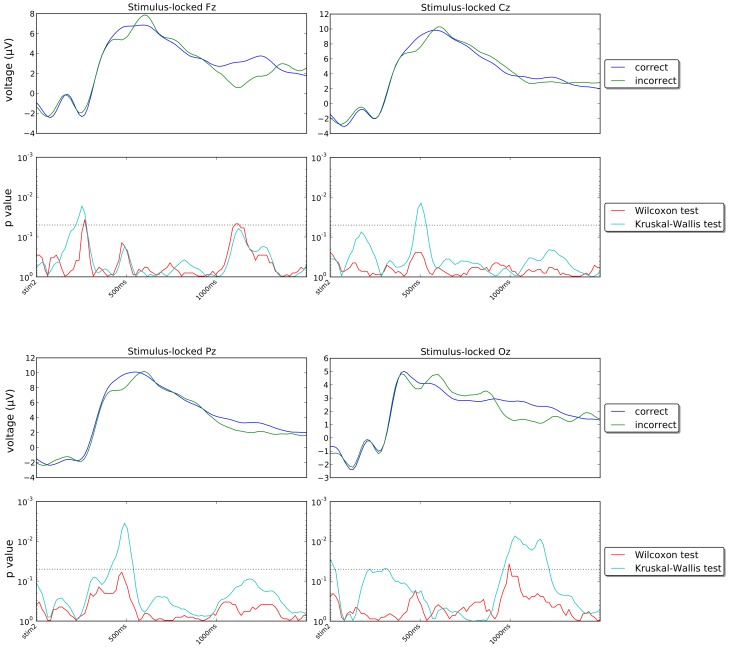
Stimulus-locked grand averages for channels Fz, Cz, Pz and Oz and corresponding temporal profile of the *p*-values of the Wilcoxon signed rank test comparing participant-by-participant averages and of the Kruskal-Wallis test for all ERPs recorded (irrespective of participant) in each error class. The dotted lines in the *p*-value plots represent the 5% confidence level. The corresponding axes are oriented so that values above that line indicate statistical significance and *vice versa*.

**Figure 11:**
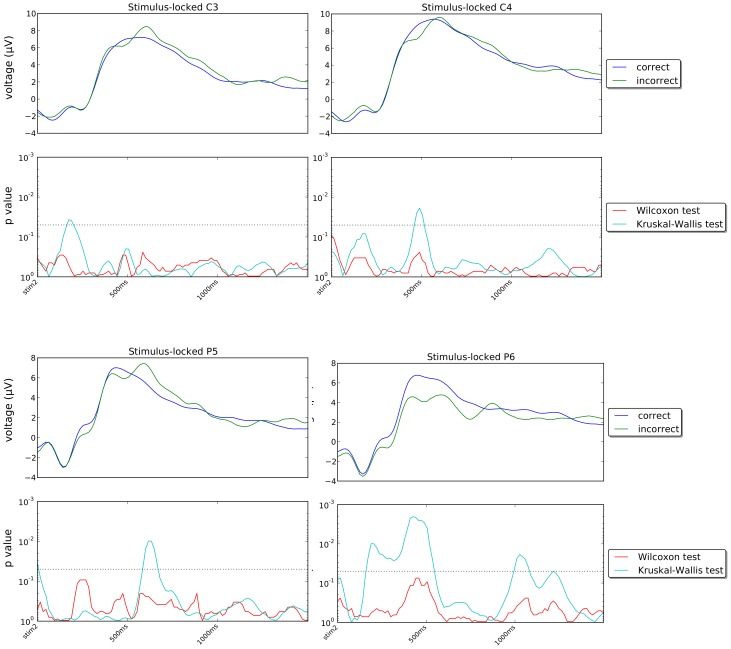
Plots of stimulus-locked grand averages and *p*-values as in **Figure 10** but for channels C3, C4, P5 and Pz (see caption of **Figure 10** and text for more details).

**Figure 12:**
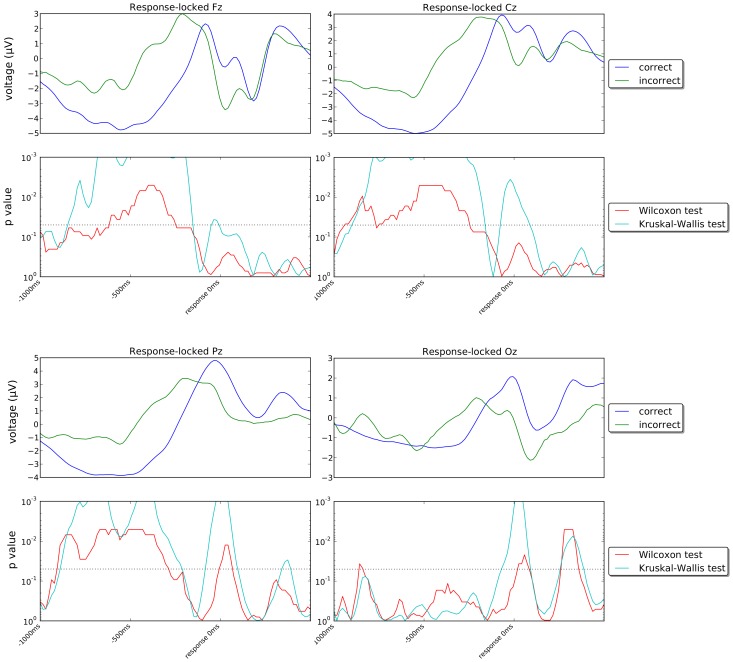
Response-locked grand averages for channels Fz, Cz, Pz and Oz and corresponding temporal profile of the *p*-values of the Wilcoxon signed rank test comparing participant-by-participant averages and of the Kruskal-Wallis test for all ERPs recorded in each error class. The dotted lines in the *p*-value plots represent the 5% confidence level. The corresponding axes are oriented so that values above that line indicate statistical significance and *vice versa*.

**Figure 13:**
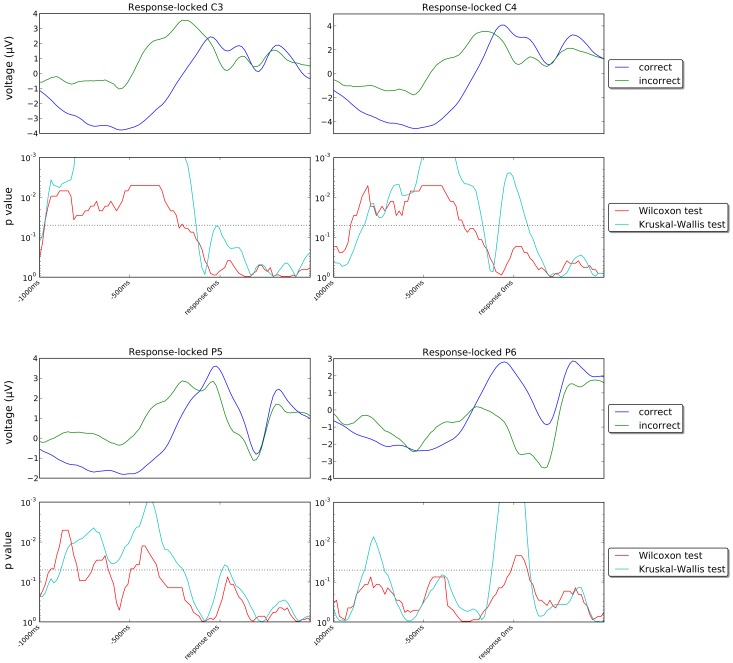
Plots of response-locked grand averages and *p*-values as in **Figure 12** but for channels C3, C4, P5 and Pz (see caption of **Figure 12** and text for more details).

Figures 10 and 11 show the stimulus-locked grand averages (averages of individual averages) of the ERPs recorded in our experiment for correct and incorrect responses for channels Fz, Cz, Pz, Oz, C3, C4, P5 and P6 (first and third rows) and the *p*-values of the statistical tests comparing the signals for correct and incorrect trials (second and fourth rows) in the period immediately following the onset of stimulus Set 2. Figures 12 and 13 show corresponding response-locked grand averages.

**Figure 14:**
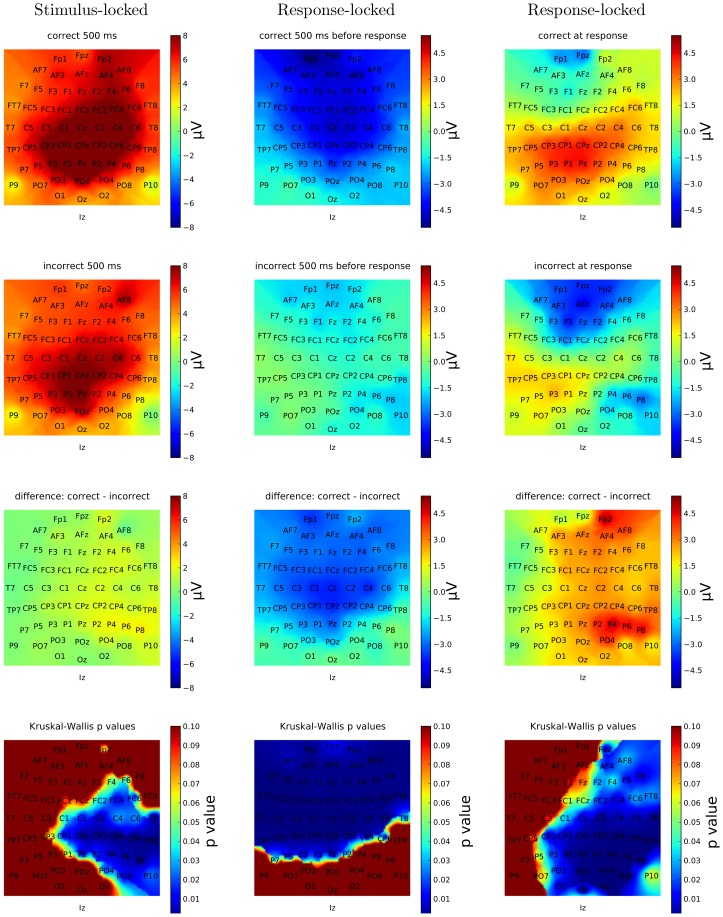
Scalp maps of the neural activity recorded 500-locked grand averages (left), and 500 ms before the response (centre) and at the response (right) as represented by the response-locked grand averages. The activity for correct and incorrect trials is depicted in the first two rows of the figure; their difference is reported in the third row; the corresponding *p*-values of the Kruskal-Wallis test are shown in the fourth row.

If we look at the grand averages in Figures 10 and 11, we see that generally there are seemingly small differences between the ERPs for correct and incorrect trials. Differences do exist, however, particularly in the region where the P300 wave peaks (approximately 500 ms after the presentation of Set 2) and for central and posterior electrodes in the right hemisphere, i.e., Cz, Pz, C4 and P4. Similar differences are present in many other channels in the same regions, as shown in Figure 14(left) which shows a snapshot of the scalp potentials recorded 500 ms after the presentation of the stimulus (in a stimulus-locked reference system).

If we look at the response-locked grand averages in Figures 12 and 13, however, we see much larger differences between the correct and incorrect responses in all 8 channels shown, either in the period preceding the response or during it or in both, with most of these differences being highly statistically significant. Similar differences are present in most other channels, as shown in Figure 14 which shows snapshots of the scalp potentials recorded 500 ms before the response (centre) and at the response (right).

We should note that a response-locked reference system amplifies the differences in the duration of the memory-retrieval and decision phases following the presentation of the stimulus for the two conditions. More specifically, P300s start approximately 600 ms before the response for incorrect decisions and approximately 400 ms before the response for correct decisions (as the corresponding median response times are approximately 880 ms and 690 ms, respectively). They peak at approximately 400 ms and 200 ms before the response, respectively. This temporal shift and the small differences in P300 amplitude seen in the stimulus-locked grand averages for the two conditions cause the large statistically significant differences observed in a response-locked reference system up to 150 ms before the response (see Figure 14(centre)).

## Conclusions

The purpose of this study was to investigate whether a collaborative BCI could be developed that would improve group decisions in a visual matching task over the performance of both a single non-BCI user and an identically-sized group of non-BCI users. The approach we have taken is unusual in relation to previous studies on collaborative BCI in that here we have exploited not only neural data but also behavioural measures of confidence to weigh group members' decisions on a decision-by-decision basis.

Experimental evidence gathered with 10 participants conclusively indicates that group decisions (whether BCI-assisted or not) are nearly always statistically significantly superior to single user decisions. Also, BCI-assisted group decisions obtained by weighting observers' decisions via our *nf*-based and *RTnf*-based methods were almost always statistically better than those obtained by equally-sized (non-BCI) groups adopting the majority rule.

We analysed the relationship between performance and response times. As predicted, we found that faster individual response times are associated with increased accuracy. We also found that the larger a group, the longer it takes to gather all the single decisions and give a group response, so that the advantage obtained by groups over a single observer in terms of accuracy is associated with a disadvantageous response time. Based on these observations, we considered a scheme where only the fastest respondents of each group influence the group's decision and found that this improves significantly the group's response time with very little or no cost in terms of accuracy, making groups not only more accurate but also faster than single observers.

As discussed in the section entitled “Decision Making in Groups”, although there are many advantages of group decision making, difficulties in communication and interaction, strong leadership and group judgement biases can sometimes be obstacles, particularly when accurate and fast decisions have to be taken. Our method achieves some of benefits of groups decisions, namely error correction and knowledge/certainty integration, without requiring intra-group communication and, thereby, avoiding some of the potential weaknesses of group decision-making.

One of the aims of our study was to develop a method based on neural features related to the decision process. As discussed in the “Introduction” section, several ERP components may be possibly used as predictive of the accuracy or confidence of one's response. We chose to include in our neural feature all ERPs in the proximity of the response (before and after it) by providing the system with a 1500 ms response-locked window of EEG starting 1 s before the response. We found that this provides reliable information on decision confidence, but in future research we will also explore other possibilities.

Finally, this study has also some limitations. In particular, here observers performed a relatively simple visual matching task, which is nowhere as complex as those carried out in realistic decision-making situations. So, in the future we will need to investigate whether the benefits of our hybrid collaborative BCI approach for group decisions also accrue with more demanding real-world scenarios, with different perceptual modalities (e.g., audio signals) and with more complex decisions. Future research will also need to clarify whether it is possible to extend our approach to decisions where different team members are exposed to different sources of information (unlike here, where they were exposed to exactly the same information).
